# More Piracy

**Published:** 1885-12

**Authors:** 


More Piracy.—The editor of the *American Medical Journal* of St. Louis, very quietly appropriates two of our original articles,and publishes them as original in his November number, without the slightest reference to the source whence he obtained them, thus apparently purposly creating the impression that they were contributed to his Journal. That is what one may call "sailing under false colors;" and is a flagrant case of that kind of piracy to which a certain class of publications were recently much addicted. We refer to "Two Cases of Persistent Vomiting in Pregnancy Relieved by Electricity," by Drs. W. T. Baird, of Albany, and R. G. Williams, of Whitney, respectively, which were written for, contributed to, and published in this Journal, in August. Another journal in copying these papers, credits them to the *American Medical Journal.* We protest against an act, as unbecoming a reputable journalist as it is unjust to ourselves and our correspondents, and we do not want to see a repetition of it. We can't afford to have others "steal our thunder" in any such way. We are proud of the fact that Texas physicians are rapidly going to the front as investigators in this field, and their successes are made known through our columns. Attention is directed to the splendid paper of Dr. Paine in this issue.

